# Supplementary material for: Binocular Integrated Visual Field Deficits Are Associated With Changes in Local Network Function in Primary Open-Angle Glaucoma: A Resting-State fMRI Study
Source: Front Aging Neurosci. 2022 Jan 13;13:744139. doi: 10.3389/fnagi.2021.744139 (PMC8792402; doi:10.3389/fnagi.2021.744139)
Supplement: Supplementary file 1 [file Data_Sheet_1.PDF]

Supplementary Material

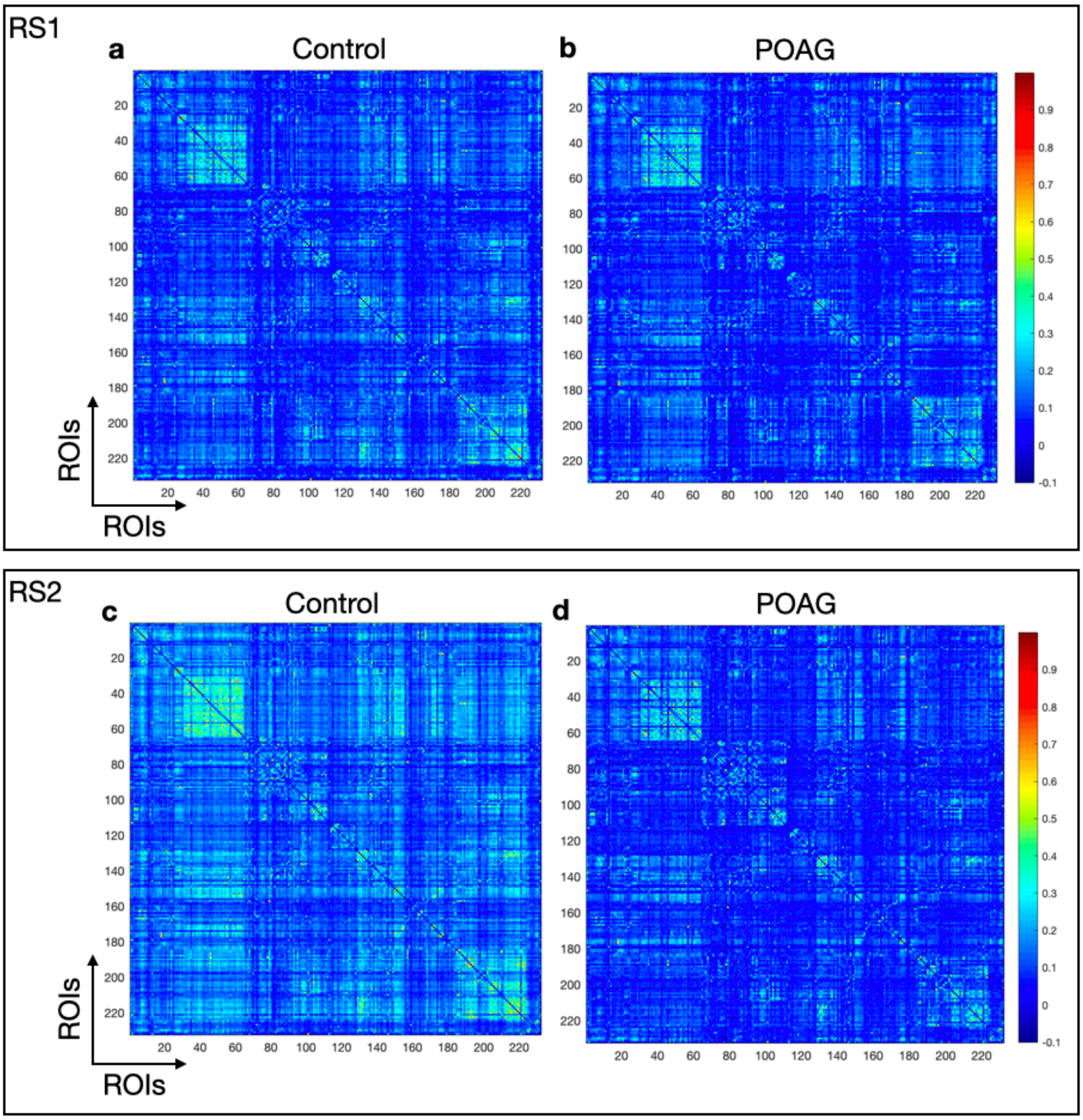

Figure S1 - Whole brain functional connectivity matrices, based on the Power atlas. For each group, first all individual functional connectivity matrices were calculated by applying Fisher's r-to-z transformation, which were then averaged across participants. Panels **a**, **b**, **c** and **d** show the mean inter-ROI correlation matrices for healthy and POAG participants, respectively.

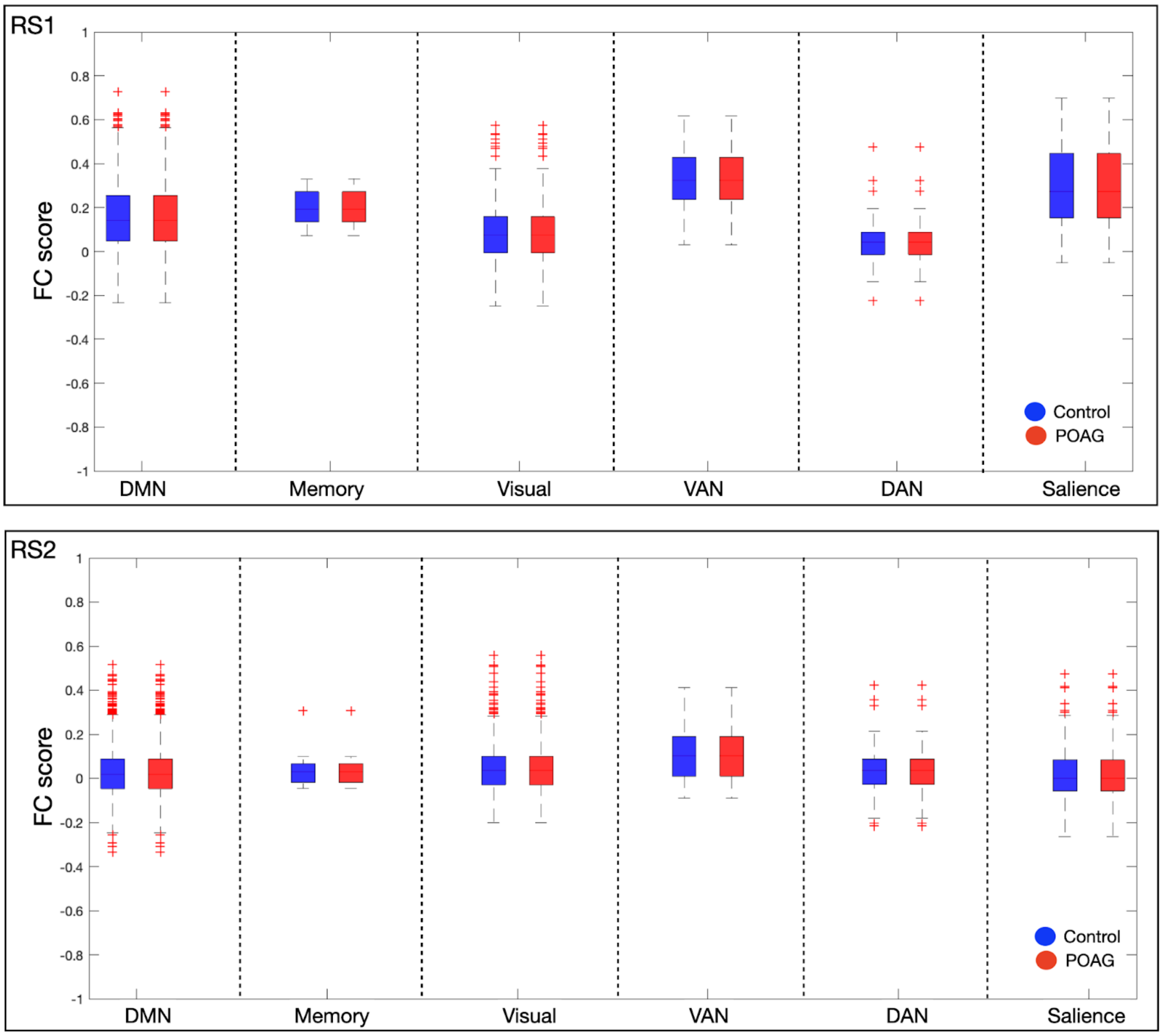

Figure S2 - Intra-group brain functional connectivity analysis based on Power atlas. For each group, we averaged the functional connectivity score across all participants for the 6 predefined Functional networks. Top panel shows the intra-group analysis for RS1 while the bottom panel reports RS2

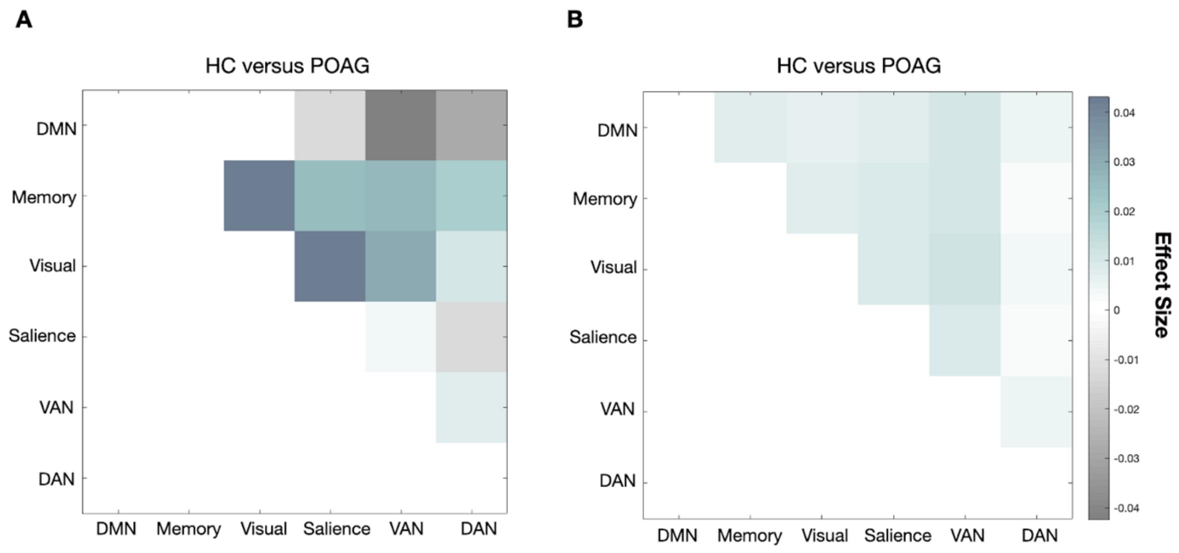

Figure S3 - Functional connectivity effect size analysis between networks. For each group, we averaged the functional connectivity score across all participants and computed the effect size between healthy and POAG groups for the 6 predefined functional networks. Panels **a** and **b** report the effect size for RS1 and RS2, respectively.

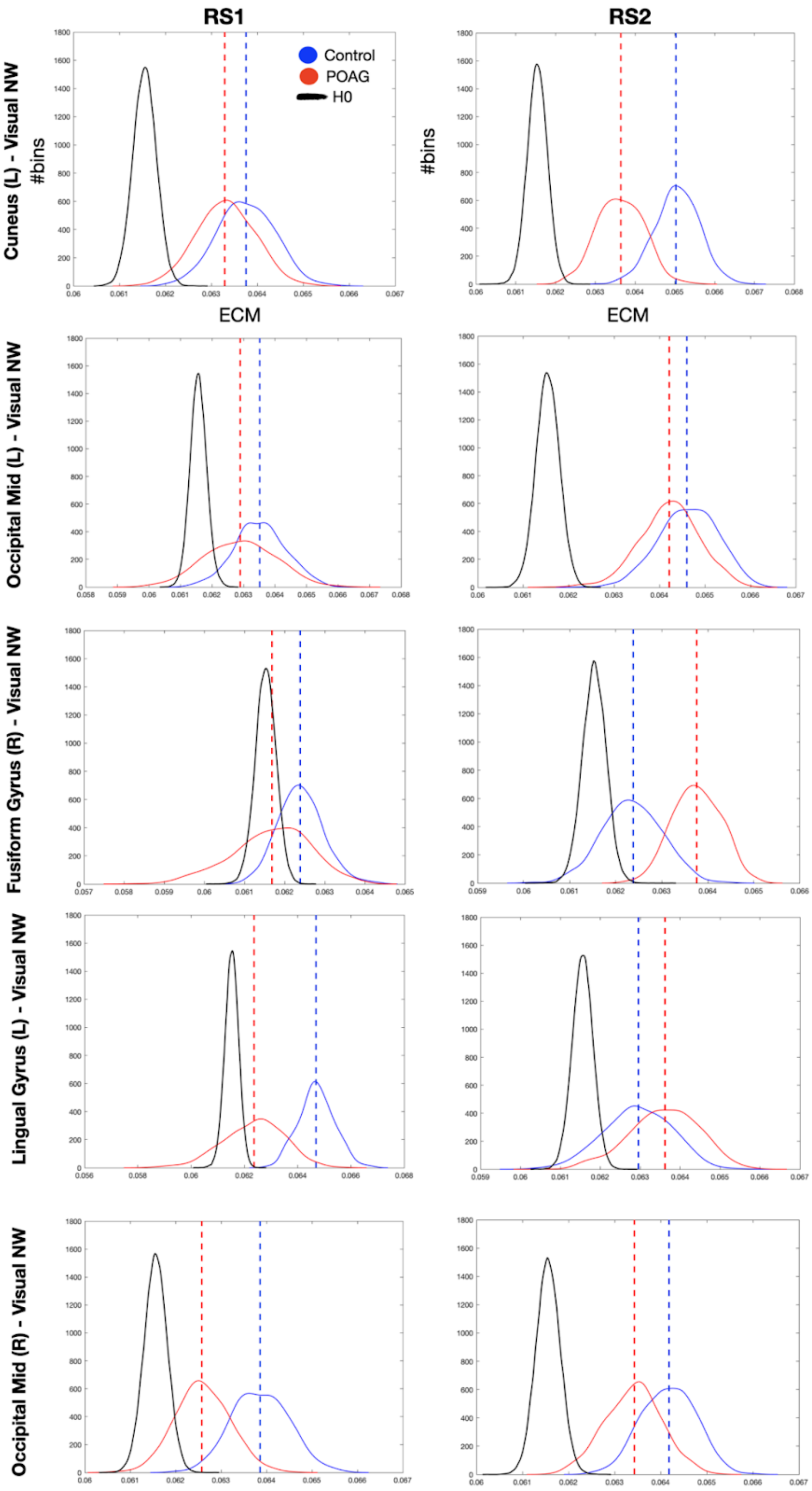

Figure S4 - EC values computed on RS1 and RS2 across groups. Eigenvector values for healthy and glaucoma groups (dashed lines), bootstrapped distributions (solid and dotted lines) and surrogate distributions (back lines) are reported for the 5% most central hubs. Interestingly,

these hubs did not significantly correlate with the behavioral scores even though they are part of the Visual Networks.

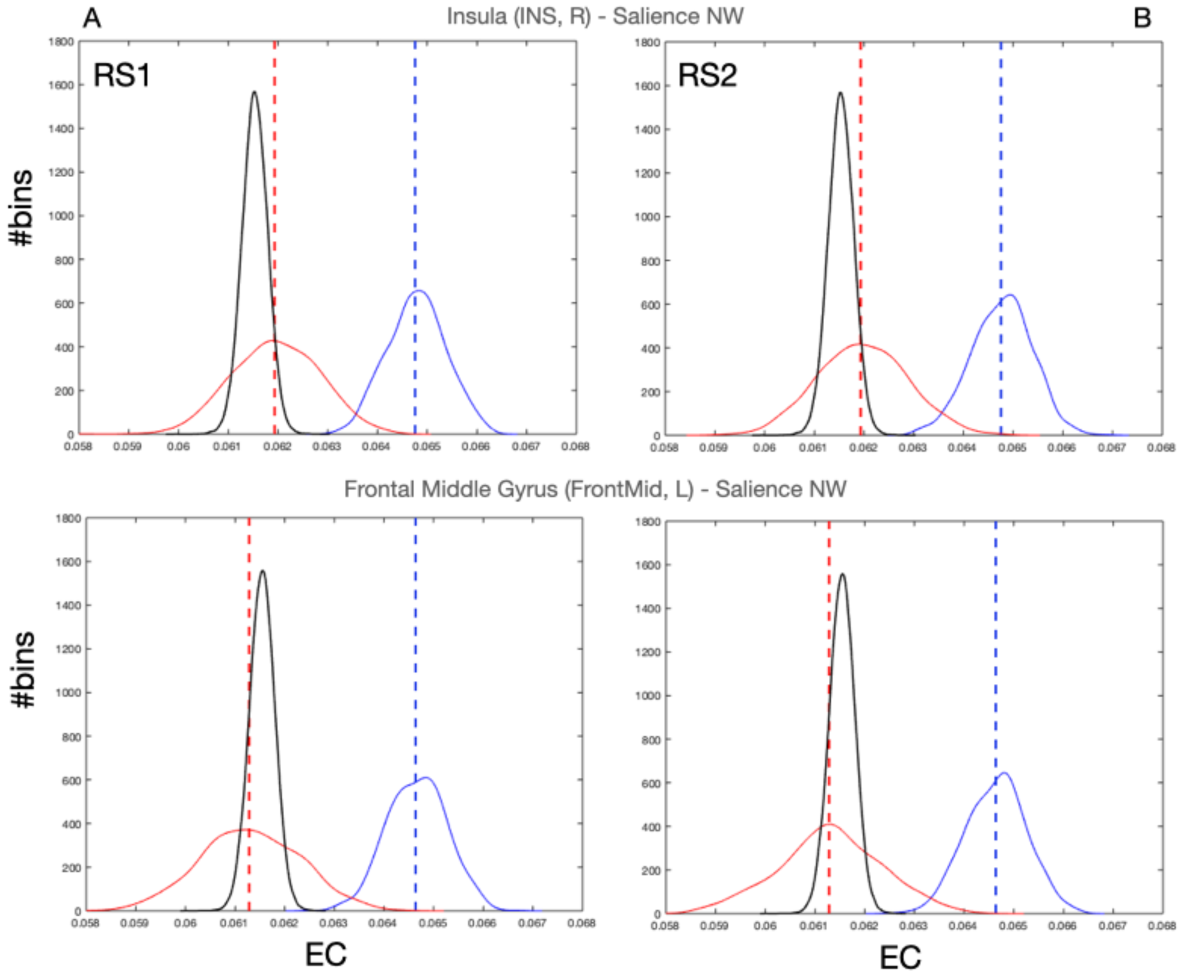

Figure S5 - EC values of the only two significant hubs present in RS2 scan. Eigenvector Centrality values of the two hubs that reached significance in RS2 scan (dashed lines), bootstrapped distributions (solid and dotted lines) and surrogate distributions for healthy and glaucoma (black lines) are reported for RS1 and RS2 in panels A and B, respectively. Note that the surrogate distributions for healthy and glaucoma participant groups are overlapping and indicated by H0 in the figure legend.

RS1

BIVF

ROIs	NW	*HC*		*POAG*		*BOTH*	
		rho	*p-values*	*rho*	*p-values*	*rho*	*p-values*
**LingG (R)**	**VIS**	0.4948	0.0435	0.293	0.2228	0.3498	0.0365
**LingG (L)**	**VIS**	-0.048	0.8548	0.0368	0.8825	0.1281	0.4565
**InfOcc (L)**	**VIS**	0.0332	0.8992	0.0509	0.8372	0.1101	0.5227
**TempSup (R)**	**VAN**	0.1637	0.5302	-0.193	0.4269	0.1509	0.3797
**FrontInfTri (R)**	**VAN**	0.1735	0.5053	-0.0491	0.8428	0.1403	0.4143
**PreCun (R)**	**DAN**	-0.2585	0.3165	0.1544	0.5266	0.1971	0.2492
**TempInf (L)**	**DAN**	0.6499	0.0047	-0.2368	0.3275	0.2379	0.1623
**MidOcc (L)**	**DMN**	0.4394	0.0776	-0.0982	0.6887	0.0359	0.8352
**FrontMedOrb (R)**	**DMN**	-0.0948	0.7175	-0.0491	0.8428	-0.2154	0.207
**RostralFrontal (R)**	**DMN**	0.2129	0.4119	0.4649	0.0465	0.0218	0.8998
**Insula (R)**	**Salience**	-0.0406	0.877	-0.0018	0.9971	-0.009	0.9584
**Insula Ant (R)**	**Salience**	-0.0726	0.7818	0.1526	0.5313	0.0386	0.9982
**MidOcc (L)**	**VIS**	-0.0135	0.9589	-0.386	0.1035	-0.245	0.1498
**Cingulate (L)**	**VIS**	-0.1415	0.5879	0.4368	0.063	0.0825	0.6323
**FrontMid (L)**	**Salience**	-0.2314	0.3715	0.3772	0.1122	0.1374	0.4243
**Fusiform (R)**	**VIS**	-0.3089	0.2276	0.0596	0.8091	-0.2517	0.1386
**MidOcc (R)**	**VIS**	0.2646	0.3047	-0.0333	0.8939	0.0878	0.6106

AbsDiffMD

ROIs	NW	*HC*		*POAG*		*BOTH*	
		rho	*p-values*	*rho*	*p-values*	*rho*	*p-values*
**LingG (R)**	**VIS**	-0.3799	0.1333	-0.2544	0.292	-0.3511	0.0358
**LingG (L)**	**VIS**	-0.4608	0.0645	-0.1105	0.6517	-0.2788	0.0997
**InfOcc (L)**	**VIS**	0.2451	0.3417	-0.2333	0.3348	-0.1525	0.3745
**TempSup (R)**	**VAN**	0.1299	0.6187	-0.1561	0.5218	-0.2147	0.2086
**FrontInfTri (R)**	**VAN**	0.0417	0.876	-0.207	0.3935	-0.152	0.3762
**PreCun (R)**	**DAN**	0.0735	0.7802	-0.1228	0.6155	-0.2451	0.1497
**TempInf (L)**	**DAN**	-0.2843	0.2678	-0.0737	0.7647	-0.1759	0.3047
**MidOcc (L)**	**DMN**	-0.049	0.8537	-0.0842	0.7318	-0.0261	0.8798
**FrontMedOrb (R)**	**DMN**	0.2794	0.2764	-0.0193	0.9397	0.2188	0.1998
**RostralFrontal (R)**	**DMN**	0.0294	0.9134	-0.0789	0.7482	0.1377	0.4231
**Insula (R)**	**Salience**	-0.4632	0.063	-0.1982	0.4142	-0.1883	0.2714
**Insula Ant (R)**	**Salience**	0.3211	0.2086	0.1404	0.5653	0.1108	0.5199
**MidOcc (L)**	**VIS**	-0.2843	0.2678	0.0684	0.7813	0.1166	0.4982
**Cingulate (L)**	**VIS**	-0.201	0.4377	0.1965	0.4184	-0.0154	0.9288
**FrontMid (L)**	**Salience**	0.4069	0.1063	-0.1175	0.6309	-0.1066	0.5362
**Fusiform (R)**	**VIS**	0.4093	0.104	0.2053	0.3976	0.3017	0.0737
**MidOcc (R)**	**VIS**	-0.1667	0.5214	0.0018	0.9971	-0.0201	0.9075

WorseMD

ROIs	NW	*HC*		*POAG*		*BOTH*	
		rho	*p-values*	*rho*	*p-values*	*rho*	*p-values*
**LingG (R)**	**VIS**	0.4951	0.0454	0.3105	0.1953	0.3426	0.0414
**LingG (L)**	**VIS**	0.0441	0.8686	0.0596	0.8091	0.1547	0.3662
**InfOcc (L)**	**VIS**	-0.0588	0.8241	0.0333	0.8939	0.0976	0.5701
**TempSup (R)**	**VAN**	0.0221	0.936	-0.1263	0.6053	0.1529	0.3719
**FrontInfTri (R)**	**VAN**	0.0515	0.8463	0.1263	0.6053	0.1606	0.348
**PreCun (R)**	**DAN**	-0.2549	0.3222	0.2211	0.3615	0.2221	0.1922
**TempInf (L)**	**DAN**	0.5319	0.03	-0.1333	0.5852	0.2291	0.1784
**MidOcc (L)**	**DMN**	0.402	0.1109	-0.1456	0.5507	0.2574	0.9994
**FrontMedOrb (R)**	**DMN**	0.0172	0.951	-0.0298	0.9053	-0.1846	0.2801
**RostralFrontal (R)**	**DMN**	0.1176	0.6526	0.4772	0.0405	-0.0299	0.8626
**Insula (R)**	**Salience**	0.0931	0.7226	0.1947	0.4227	0.0636	0.7117
**Insula Ant (R)**	**Salience**	-0.2623	0.308	-0.0281	0.9111	-0.0865	0.6148
**MidOcc (L)**	**VIS**	0.2108	0.4152	-0.2439	0.313	-0.1843	0.2808
**Cingulate (L)**	**VIS**	0.0098	0.9736	0.0263	0.9168	0.0026	0.9886
**FrontMid (L)**	**Salience**	-0.3971	0.1156	0.1895	0.4355	0.0556	0.7467
**Fusiform (R)**	**VIS**	-0.3676	0.1471	-0.0561	0.8203	-0.2798	0.0985
**MidOcc (R)**	**VIS**	0.326	0.2014	0.107	0.6622	0.148	0.3875

RS2

BIVF

ROIs	NW	*HC*		*POAG*		*BOTH*	
		rho	*p-values*	*rho*	*p-values*	*rho*	*p-values*
**LingG (R)**	**VIS**	0.1883	0.4692	0.1789	0.4619	0.3316	0.0482
**LingG (L)**	**VIS**	0.2782	0.2797	-0.2211	0.3615	-0.1082	0.5301
**InfOcc (L)**	**VIS**	0.0886	0.7352	-0.1526	0.5313	-0.09	0.6017
**TempSup (R)**	**VAN**	0.5206	0.0321	-0.0053	0.9856	0.4154	0.0118
**FrontInfTri (R)**	**VAN**	0.3274	0.1996	-0.0702	0.7757	0.3997	0.0157
**PreCun (R)**	**DAN**	0.0886	0.7352	-0.0263	0.9168	0.2306	0.176
**TempInf (L)**	**DAN**	-0.0148	0.9551	-0.3088	0.1979	-0.2159	0.2059
**MidOcc (L)**	**DMN**	0.0037	0.9888	-0.1158	0.6361	0.1929	0.2597
**FrontMedOrb (R)**	**DMN**	-0.0418	0.8733	-0.4404	0.0607	0.0635	0.713
**RostralFrontal (R)**	**DMN**	-0.0997	0.7034	0.5211	0.0238	0.3681	0.0272
**Insula (R)**	**Salience**	0.2129	0.4119	0.0228	0.9282	0.2146	0.2087
**Insula Ant (R)**	**Salience**	0.1182	0.6515	0.2754	0.2527	0.4857	0.0027
**MidOcc (L)**	**VIS**	-0.1563	0.5491	-0.0263	0.9168	-0.0901	0.6011
**Cingulate (L)**	**VIS**	-0.2548	0.3237	-0.1281	0.6003	0.0319	0.8533
**FrontMid (L)**	**Salience**	0.3569	0.1596	-0.1772	0.4664	0.1322	0.442
**Fusiform (R)**	**VIS**	-0.4849	0.0485	-0.1404	0.5653	-0.0214	0.9015
**MidOcc (R)**	**VIS**	0.4012	0.1104	0.0807	0.7427	0.1615	0.3468

AbsDiffMD

ROIs	NW	*HC*		*POAG*		*BOTH*	
		rho	*p-values*	*rho*	*p-values*	*rho*	*p-values*
**LingG (R)**	**VIS**	-0.0515	0.8463	0.6491	0.0033	-0.0687	0.6904
**LingG (L)**	**VIS**	-0.277	0.2808	-0.2368	0.3275	-0.0241	0.8892
**InfOcc (L)**	**VIS**	0.3995	0.1132	0.1175	0.6309	0.2174	0.2028
**TempSup (R)**	**VAN**	-0.0123	0.9661	0.4333	0.0653	-0.1239	0.4714
**FrontInfTri (R)**	**VAN**	0.0613	0.8167	0.6193	0.0056	-0.0898	0.6023
**PreCun (R)**	**DAN**	0.174	0.5028	-0.1632	0.503	-0.1762	0.304
**TempInf (L)**	**DAN**	0.1838	0.4786	-0.2211	0.3615	0.1292	0.4526
**MidOcc (L)**	**DMN**	-0.4167	0.0975	0.3316	0.1655	-0.1517	0.377
**FrontMedOrb (R)**	**DMN**	-0.473	0.0571	-0.2649	0.2719	-0.3057	0.0698
**RostralFrontal (R)**	**DMN**	0.5	0.043	0.386	0.1035	-0.025	0.8851
**Insula (R)**	**Salience**	-0.4779	0.0543	-0.0211	0.9339	-0.2931	0.0828
**Insula Ant (R)**	**Salience**	0.1446	0.5789	0.3772	0.1122	-0.2179	0.2017
**MidOcc (L)**	**VIS**	-0.0931	0.7226	-0.3561	0.1349	-0.1183	0.492
**Cingulate (L)**	**VIS**	-0.098	0.7084	-0.3632	0.127	-0.2986	0.0769
**FrontMid (L)**	**Salience**	-0.1642	0.5276	-0.2018	0.4058	-0.2579	0.1288
**Fusiform (R)**	**VIS**	-0.0196	0.9435	-0.0404	0.8712	-0.0802	0.642
**MidOcc (R)**	**VIS**	-0.3995	0.1132	0.0632	0.7979	-0.1474	0.391

WorseMD

ROIs	NW	*HC*		*POAG*		*BOTH*	
		rho	*p-values*	*rho*	*p-values*	*rho*	*p-values*
**LingG (R)**	**VIS**	0.2475	0.3367	-0.414	0.0793	0.2057	0.2279
**LingG (L)**	**VIS**	0.2966	0.247	0.0526	0.8316	0.8316	0.7433
**InfOcc (L)**	**VIS**	-0.076	0.7729	-0.2035	0.4017	-0.1264	0.4612
**TempSup (R)**	**VAN**	0.4387	0.0798	-0.2316	0.3386	0.3233	0.0549
**FrontInfTri (R)**	**VAN**	0.2696	0.2942	-0.4316	0.0665	0.3035	0.0723
**PreCun (R)**	**DAN**	-0.0074	0.9811	0.193	0.4269	0.234	0.1691
**TempInf (L)**	**DAN**	-0.0368	0.891	-0.0053	0.9856	-0.1282	0.4548
**MidOcc (L)**	**DMN**	0.125	0.6322	-0.2982	0.2143	0.1472	0.39
**FrontMedOrb (R)**	**DMN**	0.1593	0.5402	-0.0158	0.9512	0.2026	0.2351
**RostralFrontal (R)**	**DMN**	-0.1176	0.6526	-0.0807	0.7427	0.2064	0.2262
**Insula (R)**	**Salience**	0.326	0.2014	-0.0123	0.9626	0.2453	0.149
**Insula Ant (R)**	**Salience**	0.125	0.6322	-0.0281	0.9111	0.4095	0.0137
**MidOcc (L)**	**VIS**	0.0196	0.9435	0.3053	0.2033	0.0553	0.7478
**Cingulate (L)**	**VIS**	-0.1887	0.4668	0.1807	0.4575	0.1488	0.385
**FrontMid (L)**	**Salience**	0.375	0.1387	0.0035	0.9914	0.1905	0.2647
**Fusiform (R)**	**VIS**	-0.3505	0.168	-0.093	0.7048	0.0049	0.9779
**MidOcc (R)**	**VIS**	0.5662	0.0197	-0.0965	0.694	0.1748	0.3067

Table S1 – Correlations between behavioral scores (BIVF, AbsDiffMD, WorseMD) and EC values of identified ROIs

HC: healthy controls; POAG: primary open angle glaucoma; NW: brain network
